# Sound localization with bilateral bone conduction devices

**DOI:** 10.1007/s00405-021-06842-1

**Published:** 2021-05-06

**Authors:** Coosje J. I. Caspers, A. M. Janssen, M. J. H. Agterberg, C. W. R. J. Cremers, M. K. S. Hol, A. J. Bosman

**Affiliations:** 1 Department of Otorhinolaryngology, Donders Center for Neurosciences, Radboud University Medical Center, Nijmegen, The Netherlands; 2 Department of Biophysics, Donders Institute for Brain, Cognition and Behavior, Radboud University Nijmegen, Nijmegen, The Netherlands; 3 Department of Otorhinolaryngology/Head and Neck Surgery, University Medical Center Groningen, University of Groningen, Groningen, The Netherlands; 4 Research School of Behavioral and Cognitive Neurosciences, Graduate School of Medical Sciences, University of Groningen, Groningen, The Netherlands

**Keywords:** BAHA, BCD, Sound localization, Bilateral, Binaural hearing, Conductive hearing loss, Device use, Hearing-related quality of life

## Abstract

**Purpose:**

To investigate sound localization in patients bilaterally fitted with bone conduction devices (BCDs). Additionally, clinically applicable methods to improve localization accuracy were explored.

**Methods:**

Fifteen adults with bilaterally fitted percutaneous BCDs were included. At baseline, sound localization, (un)aided pure-tone thresholds, device use, speech, spatial and qualities of hearing scale (SSQ) and York hearing-related quality of life (YHRQL) questionnaire were measured. Settings to optimize sound localizing were added to the BCDs. At 1 month, sound localization was assessed again and localization was practiced with a series of sounds with visual feedback. At 3 months¸ localization performance, device use and questionnaire scores were determined again.

**Results:**

At baseline, one patient with congenital hearing loss demonstrated near excellent localization performance and four other patients (three with congenital hearing loss) localized sounds (quite) accurately. Seven patients with acquired hearing loss were able to lateralize sounds, i.e. identify whether sounds were coming from the left or right side, but could not localize sounds accurately. Three patients (one with congenital hearing loss) could not even lateralize sounds correctly. SSQ scores were significantly higher at 3 months. Localization performance, device use and YHRQL scores were not significantly different between visits.

**Conclusion:**

In this study, the majority of experienced bilateral BCD users could lateralize sounds and one third was able to localize sounds (quite) accurately. The localization performance was robust and stable over time. Although SSQ scores were increased at the last visit, optimizing device settings and a short practice session did not improve sound localization.

## Introduction

The percutaneous bone conduction device (BCD) is an established hearing rehabilitation method for patients with conductive or mixed hearing loss, if hearing cannot be optimized by surgery or conventional hearing aids [1]. The effectiveness of bilateral BCDs has been questioned, as due to the small intracranial attenuation one BCD will stimulate both cochleas almost equally [2, 3]. However, already in 1991, Hamann et al. demonstrated the audiological benefit of a second BCD in patients with bilateral conductive hearing loss (BCHL) [4]. Subsequently, in 1995, bilateral application of BCDs was gradually introduced. [5] Since then, several studies have shown that bilateral usage of BCDs is effective in improving speech understanding in noise [6–9], hearing-related quality of life [10–12] and sound localization in patients with BCHL [2, 6, 8, 9, 11, 13–15]. In many instances, however, patients with BCHL are still unilaterally implanted [16, 17].

Sound localization is defined as the ability to identify the direction of a sound source [18]. Sound localization is of major importance to function well in everyday life, for example, in traffic or in a crowded environment. Little is known about the actual localization performance in bilaterally aided patients [8, 9, 14]. Previous studies investigating localization in patients with BCHL used different set-ups and a limited number of loudspeakers [2, 6, 8, 9, 11, 14]. These studies either investigated whether a patient was able to lateralize sounds, i.e. identify whether sounds were coming from the left or right side [6], or evaluated whether a patient was able to localize sounds correctly within 30° or 45° [2, 8, 9, 11, 14]. In the two studies evaluating localization accuracy within 30°, 50–70% of the patients were able to localize sounds correctly [8, 9]. However, with localization accuracy being determined with 7–12 loudspeakers at 30° angles, it is unclear whether this behavior reflects localization or only lateralization. In a recently published study, a more precise sound localization test with 24 loudspeakers was used to assess localization accuracy in children with BCHL and two BCDs [11]. In that particular study, one child with acquired BCHL showed near normal localization behavior, whereas all other children were only able to lateralize sounds [13]. The one child demonstrating near normal localization, indicates that, in principle, it should be possible to localize sounds when fitted with bilateral BCDs. It would be interesting to investigate whether more (bilateral) BCD users are capable of localizing sounds, and to determine the variability in localization behavior between (experienced) users.

With sound localization being such an important feature in everyday life [19], it would be of interest to explore whether we can incorporate sound localization improving methods into our clinical practice. Improved localization might be achieved by changes in device settings and by providing localization training. However, with conventional hearing aids, it has been demonstrated that features such as compression and microphone directionality have an effect on localization performance [20, 21]. It is still unclear whether this also holds for BCDs. Furthermore, a recent study with normal hearing patients showed that localization training with visual feedback improved horizontal localization accuracy [22]. A similar training in acute monaurally deprived patients also resulted in enhanced horizontal localization [23]. 

The current study evaluated sound localization performance in 15 experienced bilateral BCD users, including patients with bilateral congenital and acquired hearing loss. In addition, we explored whether localization could be improved through optimizing device settings and a short localization practice session with visual feedback.

## Methods

### Ethical considerations

This study was approved by the local ethics committee and was conducted according to ISO14155:2011, the Good Clinical Practice guideline and the ethical principles stated by the Declaration of Helsinki [24].

### Study population

**Fig. 1:**
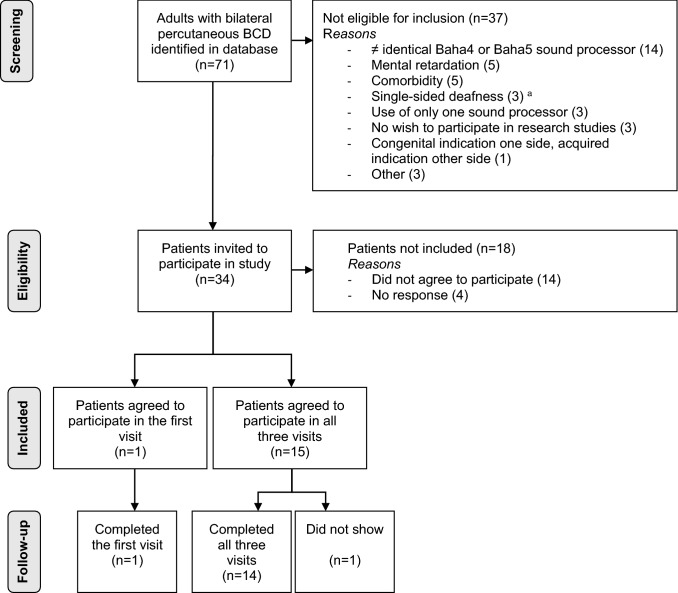
Flowchart of patient inclusion. BCD indicates bone conduction device

Fifteen adults with bilateral conductive or mixed hearing loss fitted with two identical sound processors (Cochlear™ BAHA^®^ 4 or 5) were included. Since we aimed to determine the variability in sound localization performance in our patient population, both patients with acquired and congenital hearing loss were included, as well as patients with a slight asymmetry in bone conduction (BC) thresholds. Exclusion criteria were (1) device use less than 5 days a week, (2) less than 6 months experience with bilateral BCDs and (3) inability to participate in all measurements. The patient inclusion procedure is shown in Fig. 1. Fourteen patients completed all visits and one patient completed only the first visit. Table 1 presents the characteristics of all patients. Two patients (P3 and P11) had also participated in a previously conducted study on sound localization [13]. Eleven patients had symmetric BC thresholds with an average difference at 0.5, 1, 2 and 3 kHz (PTA4) within 10 dB and individual threshold differences within 15 dB for right and left side. Asymmetric BC thresholds were found in four patients with an asymmetry in PTA4 between 11 and 13 dB (P5, P13, P15) and an asymmetry on individual frequencies between 20 and 35 dB (P5, P12, P13, P15). At the start of the study, the patients had used their bilaterally implanted BCDs for 10.4 years, on average [standard deviation (SD) 5.3] and all patients had at least 1 month experience with their current sound processors.

**Table 1:** Patient characteristics

Patient	Gender	Age	Hearing rehabilitation prior to BCD implantation	Age hearing rehabilitation (AD/AS)	Pre/post-lingual hearing impairment	Age first BCD implantation	Age second BCD implantation	Duration of bilateral BCD use (years)	Etiology	Sound processor	Localization behavior at baseline with bilateral BCDs	PTA4 AD AC	PTA4 AD BC	PTA4 AS AC	PTA4 AS BC	PTA4 aided thresholds AD	PTA4 aided thresholds AS
P1	F	60	Conventional HA ADS	18/18	post	50	51	9	COM ADS	5	Lateralization	79	33	54	28	28	29
P2^a^	F	33	Conventional HA ADS	4/4	pre	14	20	13	Microtia, atresia IIB AD Middle ear anomaly IVB AS	5	Localization	63	16	68	16	n/a	n/a
P3	M	20	B71 on headband AS	-/1.6	pre	6	6	13	Atresia IIA ADS	5	Localization	56	0	57	-3	19	16
P4	M	74	Conventional HA AD	31/-	post	63	65	9	COM ADS	5	Poor performer	71	29	73	25	24	24
P5	M	69	Conventional HA ADS	21/20	post	50	62	8	COM ADS	5	Lateralization	66	20	81	33	25	24
P6	M	75	Conventional HA ADS	45/45	post	64	69	6	COM ADS	5 p	Lateralization	71	48	76	41	34	31
P7	M	38	Conventional HA ADS	7/7	post	22	25	12	COM ADS	5 p	Localization	88	35	70	30	33	29
P8	M	69	Conventional HA ADS	32/32	post	46	47	22	COM ADS	5 p	Poor performer	65	38	48	31	23	24
P9^b^	F	44	B71 on headband AD	1/-	pre	21	24	20	Microtia, atresia ADS	4	Poor performer	64	6	68	14	26	28
P10^c^	F	50	Conventional HA ADS	6/6	pre	42	43	7	Atresia IIA ADS	5 p	Localization	55	21	46	29	23	24
P11^d^	M	18	BCD on headband	0.2	pre	7	7	12	Microtia, atresia III ADS	5	Localization	53	4	68	4	24	22
P12	F	57	Conventional HA ADS	16/17	post	33	46	11	COM ADS	5 p	Lateralization	88	31	68	29	31	34
P13	F	52	Conventional HA ADS	12/15	pre	44	48	4	COM ADS	5 p	Lateralization	81	43	59	32	30	30
P14	M	59	None	-	post	48	54	5	COM ADS	5 p	Lateralization	56	30	90	35	23	21
P15	M	66	Conventional HA ADS	34/34	post	39	61	5	COM ADS	5 p	Lateralization	62	13	58	24	27	28

For each patient the gender, age in years at study participation, type of hearing rehabilitation prior to bone conduction device (BCD) implantation (hearing aid (HA)), age at hearing rehabilitation for right (AD) and left (AS) side, pre- or post-lingual hearing impairment, age at implantation of first and second BCD, duration of bilateral BCD use in years upon study participation, etiology (chronic otitis media (COM), congenital microtia/atresia/middle ear anomaly), atresia was classified according to Cremers et al. [44], middle ear anomaly was classified according to Teunissen and Cremers [45], type of sound processor (5 *p* = 5 power), localization behaviour with bilateral BCDs at baseline, pure-tone average (PTA) at 0.5-, 1-, 2-, and 3 kHz for air conduction (AC), bone conduction (BC), and aided thresholds for right and left side are shown. n/a = not assessed

^a^P2 underwent autologous ear reconstruction for microtia on the right side at the age of 11 (performed in another institution). An exploratory tympanotomy with malleovestibulopexy on the left side was performed at the age of 16. [46] After these surgeries, conductive hearing loss worsened over time on both sides BCD implantation was performed because optimal hearing rehabilitation could not be obtained by reconstructive surgery and because of recurrent external otitis as result of the occlusion of the ear canal by the mould

^b^For P9, the classification of the atresia was unknown

^c^P10 underwent reconstructive surgery of the external auditory canal on the left side at the age of 3 and 22, and on the right side at the age of 16 and 19. BCD implantation was performed because optimal hearing rehabilitation could not be obtained with conventional hearing aids or surgery

^d^P11 used a BCD on a headband alternating between right and left side

### Study design

This study consisted of three visits: a baseline visit and two-follow-up visits at 1 and 3 months. The aim of the first visit was to determine sound localization performance in 15 experienced bilateral BCD users. The second and third visit aimed to explore whether a change in device settings and/or a short localization practice session would improve sound localization in these patients. The new BCD listening program and the short localization practice were both based on literature reports and on the expert opinion of the authors and designed to be suitable for use in clinical practice.

Baseline measures at the first visit consisted of pure-tone thresholds, sound localization performance, the speech, spatial and qualities of hearing scale (SSQ) [25], the York hearing-related quality of life (YHRQL) [26] questionnaire, device use and device satisfaction. Device use and satisfaction were assessed using the ‘daily use of bilateral BAHAs’ questionnaire [27] and a visual analogue scale (VAS), respectively. Furthermore, program usage, as logged by the sound processors, was assessed at every visit. Pure-tone thresholds were measured unaided, unilaterally and bilaterally aided. Sound localization was determined in the unilateral aided right, unilateral aided left and bilateral aided conditions. All baseline measures were performed with the patients’ habitual BCD settings. At the end of the first visit, a second listening program was added to both BCDs with settings to optimize localizing sounds by switching off adaptive microphone directionality and noise reduction, and a linear input–output characteristic by equating low- and high-level gain to the gain for 60-dB input. This fitting strategy was based on previous literature reports which observed a deterioration in localization performance when using adaptive microphone directionality and noise reduction techniques in patients with bilateral conventional hearing aids. [20, 21] Furthermore, in our experiences with bilaterally fitting conventional hearing aids, patients preferred linear amplification with a minimum set of sound processing features activated. [28] During the study, only minor gain corrections were applied upon request, while maintaining linear gain settings. Patients were instructed to use this program as much as possible.

After 1 month, to allow patients to adapt to the new listening program, a second visit was scheduled. During this visit device satisfaction and use were determined. Sound localization was again assessed in the bilateral aided condition this time with the new BCD settings. After a short localization practice session, another localization test in the bilateral aided condition was carried out with new settings. Finally, patients were instructed on explicitly using localization cues in daily life. For example, when listening with your eyes closed, guess where the sound was coming from, and subsequently open your eyes. Patients were free to follow-up on these instructions as much or little as they wanted, thus mimicking clinical practice.

At 3 months, the effects of new device settings, practice session, and instructions for daily life were evaluated by measuring sound localization with two devices with the settings to optimize the performance. In addition, the SSQ and YHRQL instruments were filled out and device use was registered. At the end of the study, device settings were set to the patient’s preference (i.e. either the original or the new settings).

### Localization test and practice session

Sound localization was measured in a sound-isolated anechoic room using the set-up described by Vogt el al [29]. In each session 75 sound stimuli were semi-randomly presented through 24 loudspeakers positioned on an arc between + 70° (right) and − 70° (left) azimuth and between + 40° (up) and − 30° (down) elevation. Loudspeakers were shielded by a black, acoustically transparent curtain. Patients were instructed to indicate the location of a sound stimulus by a head movement towards the target. Infrared cameras were used to record these head movements (Smarttrack, ART, Munich, Germany). Determining head movements is known to be an adequate method to assess localization ability [30, 31]. The 75 sound stimuli comprised 45 broadband (BB, 0.5–20 kHz), 15 high-pass (HP, 3–20 kHz) and 15 low-pass (LP, 0.5–1.5 kHz) Gaussian noise bursts. The BB stimuli were presented at 45, 55, and 65 dB SPL (15 stimuli at each sound level), whereas all HP and LP stimuli were presented at 55 dB SPL.

The practice session was performed in the same room as the localization test, with eight loudspeakers positioned in the horizontal plane at 21° apart. This 30-min practice session was performed with 65 dB SPL BB-stimuli following a stepped approach. As a first step, only six loudspeakers were used and stimuli were presented in a fixed order. Each stimulus was presented twice and the patient was instructed to listen carefully. Then, stimuli were presented randomly and the patient was instructed to indicate the position of the loudspeaker. If all responses were correct, a new task with increased difficulty was presented. In case of an incorrect response, the stimulus was presented again while providing visual feedback on the speaker position. If a patient successfully identified at least four out of the six loudspeakers, the same test was repeated with eight loudspeakers.

### Data analysis

Means (SD) and medians (interquartile range (IQR)) were used to present descriptive statistics. Localization responses were analyzed using the following criteria (1) each trial begins with a stable head position between − 10° and + 10° for at least 100 ms (ms), (2) followed by a head movement starting within 100–1500 ms after stimulus onset, ending with a stable head position, (3) stimuli are perceived within − 70° (left) and + 70° (right) azimuth. A set of 15 identical stimuli was only included for further analysis, when at least two-third of the responses met these criteria. Data analysis was performed using the approach as described by Vogt et al. [29]. Mean absolute error (MAE), response gain (slope), and bias for the best linear fit of the stimulus–response relationship were determined, separately for left and right targets. The MAE is defined as the mean of all the absolute errors, in degrees, between the position of the sound source as indicated by the patient and the actual position of the sound source. Response gain indicates accuracy, with a gain of factor 1 indicating a perfect correlation between target and response. The bias is defined as the offset in degrees. For a good performer, all data points in the stimulus–response plot will fall along the diagonal resulting in a MAE smaller than 10°, a gain close to 1 and a bias close to 0 [29, 32, 33].

Individual localization performance was evaluated using the MAE, as well as on visual assessment of the stimulus–response plots by the authors. The Wilcoxon signed rank test was used to analyze localization performance of all patients among the different listening conditions. Changes over time were analyzed with a repeated measures ANOVA (normally distributed data) or Friedman test (not normally distributed data) in case of measurements at three or more time-points, and with the paired *T*-test in case of measurements at two time-points. For correlation analysis, the Pearson correlation was used.

All analyses were performed using Matlab (MathWorks, Natick USA) and Statistical Package for Social Sciences (IBM SPSS statistics for Windows, Armonk, NY; IBM Corp, Version 25). A confidence interval (CI) of 95% was adopted and *p *value of < 0.05 was considered statistically significant.

## Results

### Sound localization

In total 60 out of 435 stimulus sets were excluded because of late responses (P5, P8), stimuli not being perceived (P6, P7, P10, P12, P13) and stimuli being perceived at the back (P4, P6, P9, P15). This mainly concerned 45 dB BB (16 data sets), HP (28 data sets) and LP (8 data sets) stimuli. Localization performance (MAE, gain and bias) with bilateral fitting at baseline did not differ significantly between sound levels or frequency bands. Therefore, localization performance is reported for BB stimuli, pooled for the three sound levels.

### Baseline performance

**Fig. 2:**
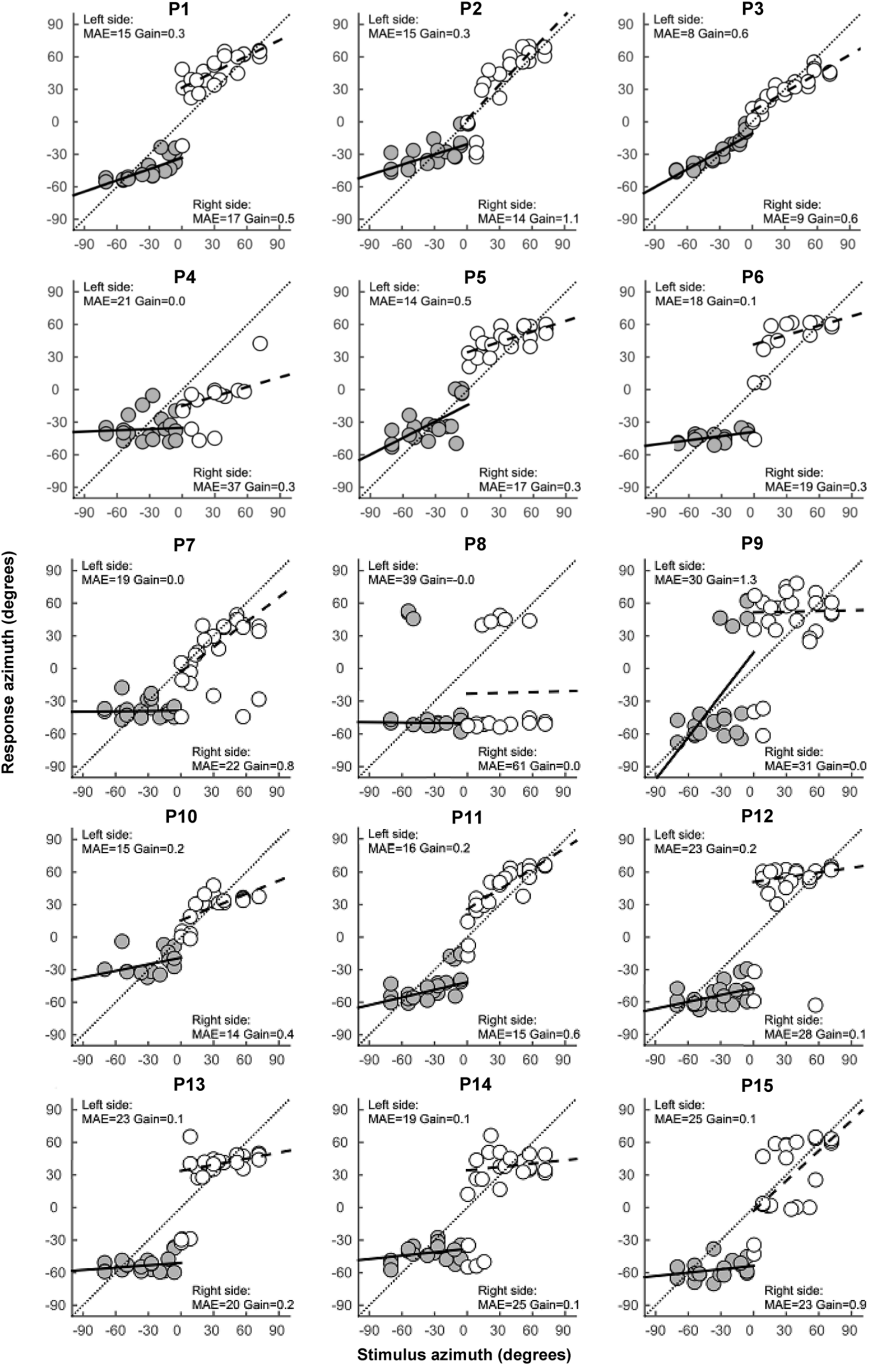
Sound localization stimulus–response plots for all patients in the bilateral aided condition at the first visit with their original device settings. The target location is plotted on the horizontal axis and the target response on the vertical axis. Negative values represent targets/responses on the patient’ left side and positive values represent targets/responses on the patient’ right side. Gray circles represent targets on the patient’ left side and white circles represent targets on the patient’ right side. For a good performer, all data points will fall along the diagonal resulting in a MAE smaller than 10° and a gain close to 1. Results are shown for broadband stimuli pooled for the 45, 55, and 65 dB presentation levels in the bilateral aided condition

Figure 2 shows the sound localization stimulus–response plots at baseline in the bilateral aided condition with the original device settings for all patients. The MAE and gain values are presented per side. In the bilateral aided condition, localization performance varied considerably among patients. In general, three performance levels were identified: (1) (quite) accurate localization, (2) lateralization only, (3) unable to lateralize sounds. In total, five patients (P2, P3, P7, P10 and P11) were able to localize sounds (quite) accurately. P3 showed near excellent localization performance (MAE < 10°) and was considered the best performing patient. P2, P10 and P11 were able to localize sounds to some extent, but not as good as P3 (MAE 14°–16°). P7 was able to localize sounds quite accurately on the right side, but not on the left side. On visual inspection of their stimulus–response plots, we classified P1 and P5 as lateralizers, despite their relatively small MAEs. Including P1 and P5, a total of seven patients were found to be capable of lateralizing sounds (P1, P5, P6, P12–15). The remaining three patients (P4, P8, P9) were poor performers unable to lateralize sounds correctly, although P9 did seem capable of lateralizing sound stimuli which were presented at more than 30° off-center.

Out of the five patients with bilateral congenital hearing loss, four patients (P2, P3, P10, P11) were able to localize sounds (quite) accurately and the remaining patient (P9) was considered a poor performer not capable of lateralizing sounds. Interestingly, localization performance differed per side in P2 and P11. All five patients with a congenital etiology had a symmetric hearing loss with normal (P2, P3, P9, P11) or near normal (P10) BC thresholds. Three of these patients (P3, P9, P11) were rehabilitated with one hearing device on a headband already as a young infant. Bilateral hearing rehabilitation was achieved between the age of 4 and 7 in four patients (P2, P3, P10 and P11) and at the age of 24 in one patient (P9). The patients who were bilaterally rehabilitated during childhood showed (quite) accurate sound localization, whereas the patient P9 in whom bilateral rehabilitation was conducted at the age of 24 was considered a poor performer.

Out of the 10 patients with bilateral acquired hearing loss, one patient was able to localize sounds quite accurately (P7), seven patients were capable of lateralizing sounds (P1, P5, P6, P12-15) and two patients were not even able to lateralize sounds (P4, P8) and therefore considered as poor performers. This group of patients with an acquired hearing loss comprised both patients with mild asymmetric BC thresholds and patients with symmetric BC thresholds of 25 dB and worse. BCD implantation was performed sequentially in all patients with an acquired hearing loss with a mean of 7 years (SD 6.6) between first and second implantation. P7 had BC thresholds of 30 and 35 dB and could localize sounds quite accurately on the right side, but not on the left side. The patients capable of lateralizing sounds had BC thresholds ranging from 14 to 43 dB and the poor performers had BC thresholds ranging between 25 and 38 dB. All four patients with asymmetric BC thresholds were capable of lateralizing sounds.

**Fig. 3:**
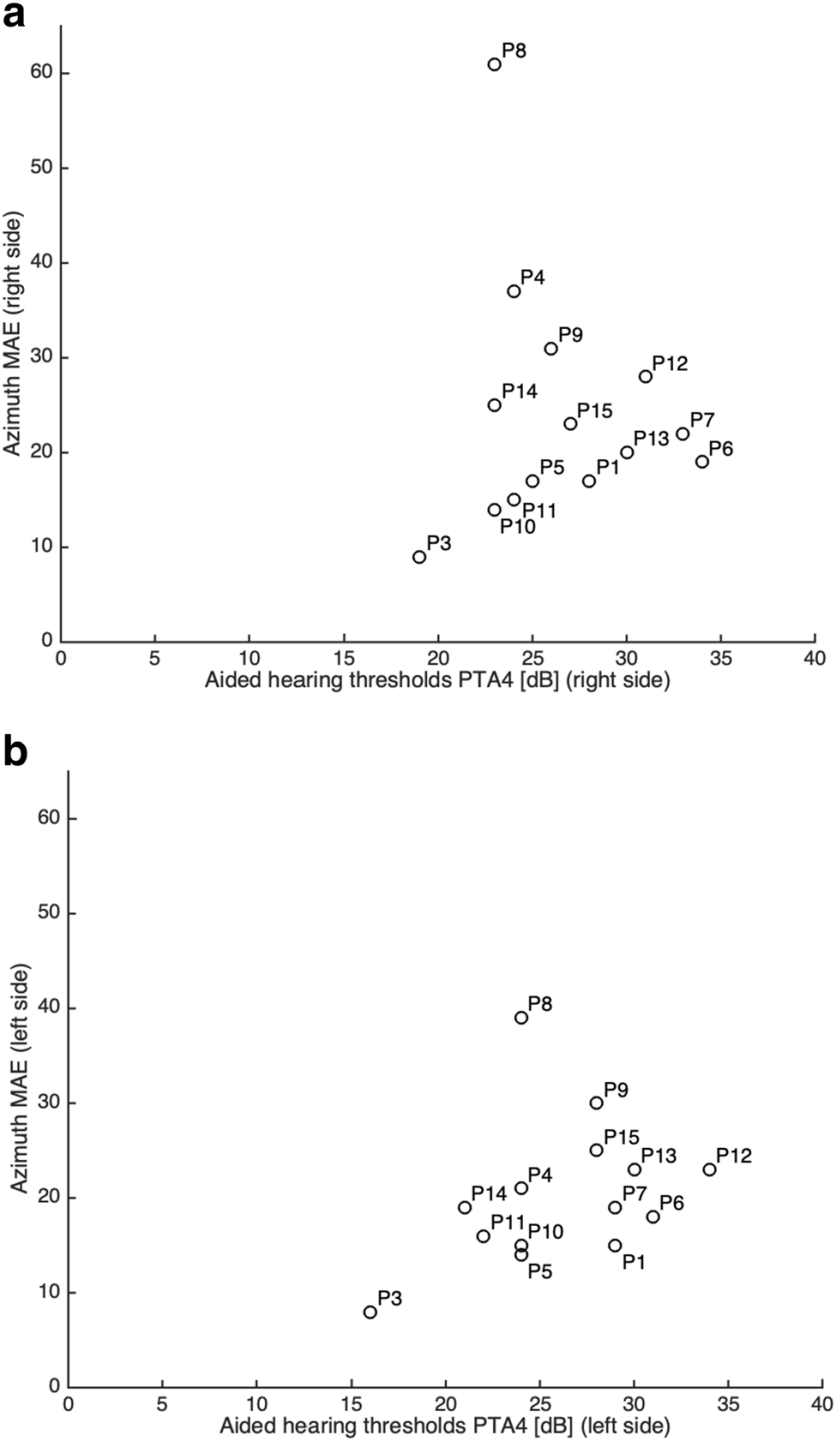
Response azimuth mean absolute error (MAE) for broadband stimuli in the bilateral aided condition at baseline, plotted against the aided PTA4 (mean of 0.5-, 1-, 2- and 3 kHz) for right side (**a**) and left side (**b**). P2 is not included in these figures since aided thresholds were not assessed in this patient

Figure 3 shows the correlation between the MAE in the bilateral aided condition at baseline and aided thresholds, for each side separately. The scatter plots suggest that the MAE, and thus localization performance, deteriorates with poorer aided thresholds. P8, a poor performer, was identified as an outlier and removed from further analysis. Pearson correlation showed a significant positive correlation between MAE and aided thresholds for the left side (*r* = 0.60, *p* = 0.03), but the correlation for these parameters on the right side was not significant (*r* = 0.25, *p* = 0.42). The correlation between BC thresholds and MAE was not significant.

### Bilateral versus acute unilateral fitting

**Fig. 4:**
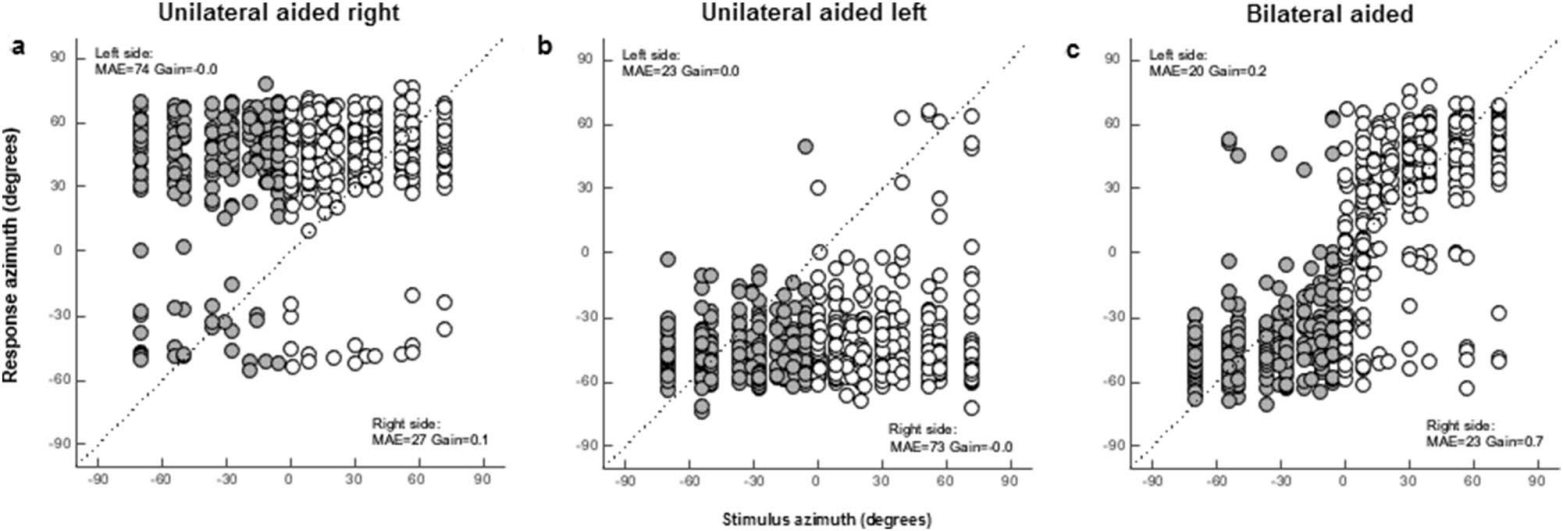
Sound localization stimulus–response plots pooled for all patients in the unilateral aided right (**a**), unilateral aided left (**b**) and bilateral aided (**c**) condition at the first visit. Gray circles represent targets on the patient’ left side and white circles represent targets on the patient’ right side. Results are shown for broadband stimuli pooled for the 45, 55 and 65 dB presentation levels

With an acute unilateral fitting, patients perceived sounds mainly on the aided side (Fig. 4). Bilateral fitting significantly improved localization performance compared to the unaided side in the unilateral aided conditions [MAE (Δ median = − 58, *p* = 0.000), gain (Δ median =  + 0.2, *p* = 0.003) and bias (Δ median = − 15, *p* = 0.000)], as well as compared to the aided side in the unilateral aided situations [MAE (Δ median = − 4, *p* = 0.007), gain (Δ median =  + 0.2, *p* = 0.000) and bias (Δ median = − 14, *p* = 0.000)]. P4 was excluded from this analysis due to insufficient reliable data points in the unilateral aided conditions. In P8, bilateral fitting did not improve performance, as stimuli were still mainly perceived on the left side. Interestingly, P8 had slightly worse BC and aided thresholds on the right side, and only perceived 65 dB stimuli while wearing one device on the right.

### Localization performance between visits

**Fig. 5:**
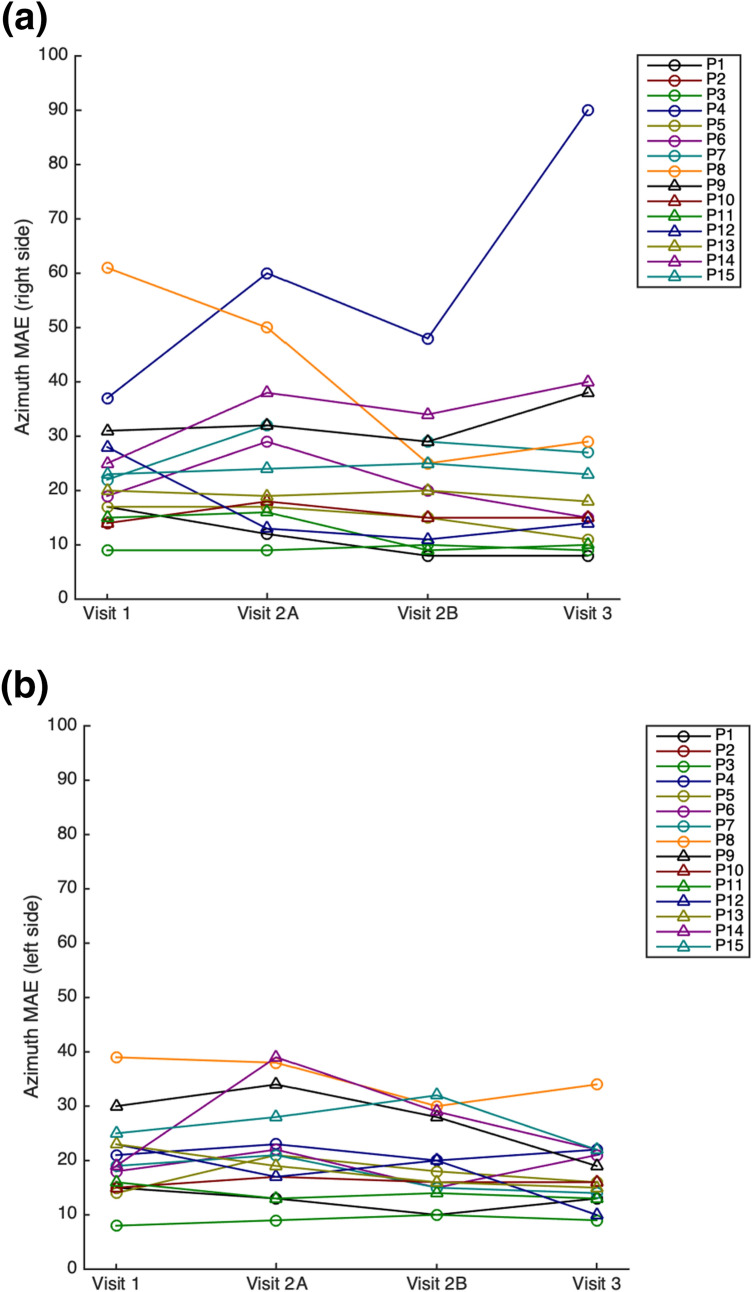
Line graph showing the azimuth mean absolute error (MAE) of broadband stimuli among the four localization tests in the bilateral aided condition, separate for right side (**a**) and left side (**b**). Visit 1 represents baseline measurements, visit 2A the measurement after device settings were changed and visit 2B the measurement after the localization practice session with visual feedback. At visit 3, localization was measured to evaluate the combined effects of device settings and the localization practice session. P2 only participated in the first visit and was therefore not included in these figures

On a group level, statistical comparisons of the MAE, gain and bias did not reveal any consistent significant differences between visits. In line with this, Fig. 5 demonstrates that for most patients MAE values were quite similar across the four localization tests in the bilateral aided condition. For a few patients however, MAE values did differ among visits (for instance in P4 and P8, Fig. 5a). These differences in MAE values were, however, not consistent over time and differed per side.

### Device use, satisfaction and hearing-related quality of life

At baseline, all patients reported to be either very satisfied (66.7%) or satisfied (33.3%) with their BCDs with a median VAS of 8.1. All patients used both sound processors simultaneously at all times. Fourteen patients used them more than 12 h a day and one patient (P8) between 4 and 8 h a day. Device use or device satisfaction did not change significantly between visits. Between the first and second visit, median device use was 15 h/day (IQR 12–15). The newly added listening program with settings to optimize localization was used most of the time (median percentage of the total wearing time 92%, IQR 80–99%). At the end of the study, 13 patients (87%) preferred the new device settings, specifically because of improved clarity and loudness of sounds. At the third visit, a significant improvement in SSQ scores was found for the subdomains spatial hearing (+ 1.5, *p* = 0.001) and quality of hearing (+ 1.0, *p* = 0.006), and for the total score (+ 0.97, *p* = 0.001). Scores for the SSQ subdomain speech and understanding, and YHRQL did not differ significantly between visits.

## Discussion

In this study, the majority of patients with bilateral conductive hearing loss (BCHL) fitted with bilateral bone conduction devices (BCDs) was able to lateralize sounds at baseline (i.e. already during the first visit). Interestingly, bilaterally fitted patients with bilateral acquired as well as patients with bilateral congenital hearing loss, were capable of localizing sounds (quite) accurately. This finding differs from previous research in which localization behavior of both congenital and acquired hearing-impaired bilateral BCD users, was limited to lateralization (i.e. localization within 30° of the correct speaker location) [8, 9]. These differences in localization performance might be a result of differences in study design. Whereas in our study, the mean absolute error (MAE) was used to describe localization performance, Priwin et al. and Bosman et al. determined correct responses on the precise target location, and correct responses within 30° of the target location [8, 9]. In the current study, localization performance was thus described more precisely, and to our knowledge this is the first study reporting a robust and stable localization performance (i.e. no variation in performance over time). In the study of den Besten et al. the MAE was also used to determine localization performance with bilateral BCDs. In that particular study, all children with bilateral congenital conductive hearing loss were only able to lateralize sounds [13]. The one child able to localize sounds had a bilateral acquired hearing loss. Interestingly, in the current study, both patients with acquired and congenital hearing loss were capable of (quite) accurate sound localization. Based on the current studied patient population, localization accuracy is not necessarily related to the time of onset of hearing loss. The question remains how to explain the variability in sound localization performance in patients with bilateral BCDs, even within groups of patients with the same etiology. Possible explanations are the variety in age at study participation, bone conduction (BC) thresholds, BC asymmetry and age of bilateral hearing rehabilitation [34, 35]. Unfortunately, an extensive statistical exploration of the effect of these characteristics on sound localization could not be performed due to the small sample size of our study population. However, some interesting observations were made.

First of all, sound localization was more accurate in patients with symmetric and near normal bone conduction thresholds when compared to patients with either asymmetric BC thresholds or patients with BC thresholds of 25 dB and higher. This might suggest that reasonable localization scores can only be obtained with symmetric, near normal BC thresholds. On the other hand, near normal symmetric thresholds do not warrant good localization performance as one patient with normal, symmetric BC thresholds was not even able to lateralize sounds.

Second, in this study, patients with a limited asymmetry in BC thresholds, were still able to identify whether sounds were coming from the right or left side when using bilateral BCDs. In general, sound localization is known to deteriorate with increasing BC asymmetry [36]. However, little information on exact localization performance in patients with asymmetric bone conduction thresholds and bilateral BCDs is available. Based on the current study, patients with a limited asymmetry seem capable of lateralizing sounds correctly but are not able to localize sounds more precise than that.

Third, two of the patients with congenital hearing loss (P3 and P11) had already participated in another study on localization as a child. In that study with a similar test set-up, the MAEs with bilateral devices were 45 (P10) and 36 (P9) respectively [13]. In the current study, MAEs of 9 (P3) and 16 (P11) were found for the worst performing side of these patients. The improved MAEs in the current study might be explained by increased experience with bilateral BCDs, by an increasing age or by differences in measurement protocols.

Finally, our findings suggest that a period of hearing rehabilitation with one device in patients with bilateral congenital conductive hearing loss does not necessarily rule out fairly accurate localization scores with bilateral fitting in early and middle adulthood [9]. Yet, to achieve this fairly accurate localization ability, bilateral hearing rehabilitation should probably be realized before late childhood, since the main developments in auditor discrimination in normal hearing children take place between 6 to 7, and 8 to 9 years old [37]. Unfortunately, it is not possible to define a precise age cut-off point for bilateral rehabilitation, since it is still unclear at what age spatial processing abilities are fully developed. The development of more specific skills, such as discrimination of stimulus frequency, intensity and duration is thought to continue past this age range [37, 38]. Furthermore, maturation of the auditory cortex and growth of the head circumference continue until adolescence and adulthood, respectively [39, 40]. Therefore, the four patients in our study who were bilaterally rehabilitated during childhood (i.e. ≤ 8 years of age) might have been able to develop binaural hearing skills within this time window, thus showing (fairly) accurate localization performance.

At our tertiary referral center, bilateral hearing rehabilitation with two BCDs on a softband as early as possible and consecutively simultaneous bilateral percutaneous implantation from the age of 4, is provided in children with bilateral congenital conductive hearing loss as standard of care since 2009 [27]. The rationale behind this practice is the proven benefit of bilateral BCDs in terms of speech understanding in noise [6–9], hearing-related quality of life [10–12] and sound localization in patients with BCHL [2, 6, 8, 9, 11, 13–15]. Furthermore, the current study implies that early bilateral hearing rehabilitation enhances localization skills at a later age. We believe it is of importance to optimize hearing performance as early as possible to ensure adequate speech- and language development. Consequently, we recommend bilateral rehabilitation is performed at a subsequent stage, from the age of four. For a future study, it would be interesting to determine sound localization performance in a larger group of adult patients who underwent early bilateral hearing rehabilitation as a child. Such research might confirm the suggested importance of early bilateral revalidation for developing localization abilities.

### Improving localization

Even though our customized device settings did not clearly improve localization performance in this study, we would yet suggest to provide this new setting protocol to all bilateral BCD users. The rationale is that the large majority of patients preferred this setting because of its clarity and loudness. A localization practice session suitable for clinical practice was not found to improve sound localization in a group of experienced BCD users. However, scores on the speech, spatial and qualities of hearing scale did improve at the last visit. Our practice session might have been too short to have an effect on localization performance in this group of experienced users. Also, baseline grades for device satisfaction and sound processor use were already high in this population. This raises the question whether (further) improvement of localization skills is feasible in experienced and satisfied patients. On the other hand, localization training has been effective in other types of hearing-impaired patients [23, 41] and not all our patients were capable of localizing sounds. We believe it remains important to further determine factors influencing localization ability, to develop efficient methods for improved localization with bilateral BCDs.

### Strengths and limitations

This is the first study into evaluating the efficacy of adjusting device settings, and of practicing with visual feedback, on sound localization in patients fitted with bilateral BCDs. Current literature only describes device settings and training in normal hearing patients [22], unilateral hearing-impaired patients [23] or bilateral hearing-impaired patients using conventional hearing aids [41, 42]. Therefore, both the listening program with settings to optimize sound localization and the practice session were mainly based on expert opinions. During the localization tests, some patients did not perceive all sound stimuli. Additionally, some patients perceived stimuli at the back, whereas sound stimuli were only presented in the frontal plane. The latter finding is probably a result of front-back confusion due to the absence of pinna cues [43]. For future studies, we would therefore recommend to present broadband sound stimuli of sufficient intensity within an arc of 360°. Another possible limitation is that only regular BCD users were included in this study and therefore all patients were (very) satisfied with their devices and used both devices on a regular basis. Localization performance of unsatisfied or non-regular users might differ from the findings in our study. Also, localization performance in the unilateral aided conditions represents an acute condition, as all patients were not accustomed to listening with only one device. So, performance in the unilateral aided condition might differ from patients with BCHL who are accustomed to wearing one device.

## Conclusion

In patients with bilateral conductive hearing loss, a second bone conduction device (BCD) seems to improve localization performance. The majority of patients fitted with two BCDs could distinguish whether sounds were coming from the left or right side (lateralizing behavior), and one third of patients was able to localize sounds (quite) accurately. All patients with a slightly asymmetric hearing loss were capable of lateralizing sounds. Localization performance was stable over time. Although scores on the speech, spatial and qualities of hearing scale did increase at the last visit, a listening program tailored for localizing sounds and a short localization practice session did not improve localization performance on a group level. More research into the variability in localization performance as well as methods for further improving localization skills in patients with two BCDs is warranted.
